# External ear malformations and cardiac and renal anomalies: A systematic review and meta-analysis

**DOI:** 10.1371/journal.pone.0309692

**Published:** 2024-09-19

**Authors:** Arman Ghafari, Leonardo Alaniz, Cindy Vu, Alejandra Ibarra, Medha Vallurupalli, Sierra Willens, Justin Cordero, Miles J. Pfaff

**Affiliations:** 1 University of California Irvine, School of Medicine, Irvine, CA, United States of America; 2 Keck School of Medicine of USC, Los Angeles, CA, United States of America; 3 Stanford University School of Medicine, Palo Alto, CA, United States of America; 4 Department of Plastic Surgery, University of California Irvine Medical Center, Orange, CA, United States of America; 5 University of California Riverside, School of Medicine, Riverside, CA, United States of America; Shaheed Rajaei Hospital: Rajaie Cardiovascular Medical and Research Center, ISLAMIC REPUBLIC OF IRAN

## Abstract

**Context:**

External Ear Malformations (EEM) continue to be a common malformation seen in the pediatric patient population. This study aims to further elucidate the correlation between EEM and cardiac and renal anomalies.

**Objective:**

A systematic review and meta-analysis to study the incidence of cardiac and renal anomalies associated with syndromic and isolated (EEM).

**Data sources:**

The literature search spanned multiple databases, including Google Scholar, PubMed, Scopus, Web of Science, and MEDLINE.

**Study selection:**

Studies must be focused on EEM and cardiac and/or renal anomalies. Only articles written in English were included.

**Data extraction:**

General study characteristics, number of EEM patients, number of cardiac and renal anomalies and whether cases were syndromic were extracted from the studies.

**Results:**

Of 1,058 initial studies, 33 were included for meta-analyses. Mean JBI score for all included studies was 92.06%, indicating acceptable study quality. Interrater reliability was high, with a Cohen kappa score for all studies of 0.94. The resulting pooled prevalence of cardiac abnormalities was 20% [95% CI:13–28%], while renal abnormalities were 13% [95% CI: 7–20%]. The most common anomalies were VSD (3.725%) and renal agenesis (2.04%). The presence of syndrome data across studies was not a significant modifier of prevalence rates.

**Limitations:**

Primary limitation is due to heterogeneity in individual study methodology and reporting standards.

**Conclusions:**

These results highlight a higher prevalence of cardiac-related conditions than renal anomalies in patients with both syndromic and non-syndromic EEM in the included studies, underscoring the need for thorough clinical evaluations.

## Introduction

External ear malformations (EEM) encompass a spectrum of congenital craniofacial anomalies including microtia, accessory tragus, preauricular sinus and pits that are encountered in clinical practice [1]. These malformations are traditionally recognized for their aesthetic and structural implications but may also represent a broader clinical significance given the potential association with systemic pathologies, specifically with cardiac and renal anomalies.

The literature presents a fragmented landscape regarding the prevalence and characterization between EEM and cardiac and renal anomalies. An in-depth understanding of such associations could optimize clinical outcomes through early detection and intervention and foster interdisciplinary collaboration, enriching patient care strategies. This systematic review and meta-analysis strives to characterize the nature and frequency of renal and cardiac anomalies concomitant with external ear malformations, aiming to inform and refine clinical assessment protocols.

## Materials and methods

### Literature review

This systematic review and meta-analysis was exempt from review by the institutional review board and was performed in accordance with the Preferred Reporting Items for Systematic Reviews and Meta-Analyses (PRISMA) [2] and was registered with PROSPERO (ID # CRD42023434894) after ensuring no prior similar studies had been published. EEM was defined by a range of congenital craniofacial anomalies that affect the external ear structure, including but not limited to microtia, anotia, preauricular pits, preauricular sinus, and accessory tragus. A literature search was conducted on July 5^th^, 2023, using a predetermined Boolean query in the following databases: Google Scholar, PubMed, Scopus, Web of Science, and MEDLINE. A list of medical Subject Headings (MeSH) terms and keywords were developed for ear malformations, cardiac abnormalities, and renal abnormalities; search strings are presented in S1 Table. Publication dates ranged from inception to 2022. All articles were imported into Covidence, an online data extraction tool for systematic reviews [3].

### Eligibility criteria

Inclusion criteria included studies that demonstrated reportable outcomes describing EEM and renal and cardiac abnormalities. Larger epidemiological studies utilizing national databases and other systematic reviews were excluded to prevent duplication of data. Only articles written in English were included. Exclusion criteria included studies with sample sizes of less than five patients, articles that did not demonstrate a focus on any associations of interest, and/or did not contain quantitative data. The articles selected for inclusion underwent a rigorous review process, conducted by a team of four independent reviewers (AG, AI, CV, and MV). This process began with an initial screening based on the title and abstract of each article, followed by a detailed evaluation of the full text. To ensure the highest level of scrutiny and objectivity, each study was meticulously evaluated twice at both initial and full-text stages, ensuring an unbiased assessment. Any disagreements between reviewers for article inclusion were resolved by a third-party reviewer (MJP). Methodologic quality was assessed for each study using Joanna Briggs Institute (JBI) [4] scoring by two independent scorers, and interrater reliability was measured using Cohen’s kappa.

### Data extraction

Data extracted included the following: country in which the study was conducted, study design, level of evidence (LOE), study start and end date, sample size, patient demographics, type of EEM, type of renal abnormality, type of cardiac abnormality, associated disorders or syndromes, and imaging modality used for evaluation.

### Data analysis

Initially, the raw rates of cardiac and renal anomalies in patients with EEM were extracted from each study and adjusted to calculate weighted prevalence rates. This adjustment was done by proportionally scaling the data from each study according to its sample size relative to the total patient population across all included studies. Given the observational characteristic of this study, proportion meta-analyses were conducted to obtain a pooled prevalence of renal and cardiac anomalies in EEM patients. Double arcsine transformations were applied to the observed proportions to construct a normal distribution pattern and facilitate the estimation of summary proportions. A random-effects model based on the DerSimonian-Laird inverse variance estimator method was implemented to account for the observed variability in populations and methodologies across all studies and provide a conservative summary estimate. After the analysis, estimates were converted back to the original scales to yield interpretable summary proportions. Q-statistic, I^2^, and *τ*^*2*^ were calculated to identify and quantify heterogeneity across all included studies and guide balanced weight assignment. A Q-statistic value of p<0.10 and I^2^ values greater than 50 percent indicated significant heterogeneity. Post-hoc sensitivity analyses were conducted to identify outlying effect sizes by screening for externally studentized residuals and subsequently conducting “leave-one-out" tests to identify overall summary proportion changes. Thereafter, binary moderator tests were conducted investigating the effect of study year, sample size, and presence or absence of syndrome data. Lastly, subgroup analyses were conducted on microtia cases (86% of included cases) versus all other EEM cases. Statistical analyses were performed using R for Macintosh 2023 [5].

## Results

**Fig 1:**
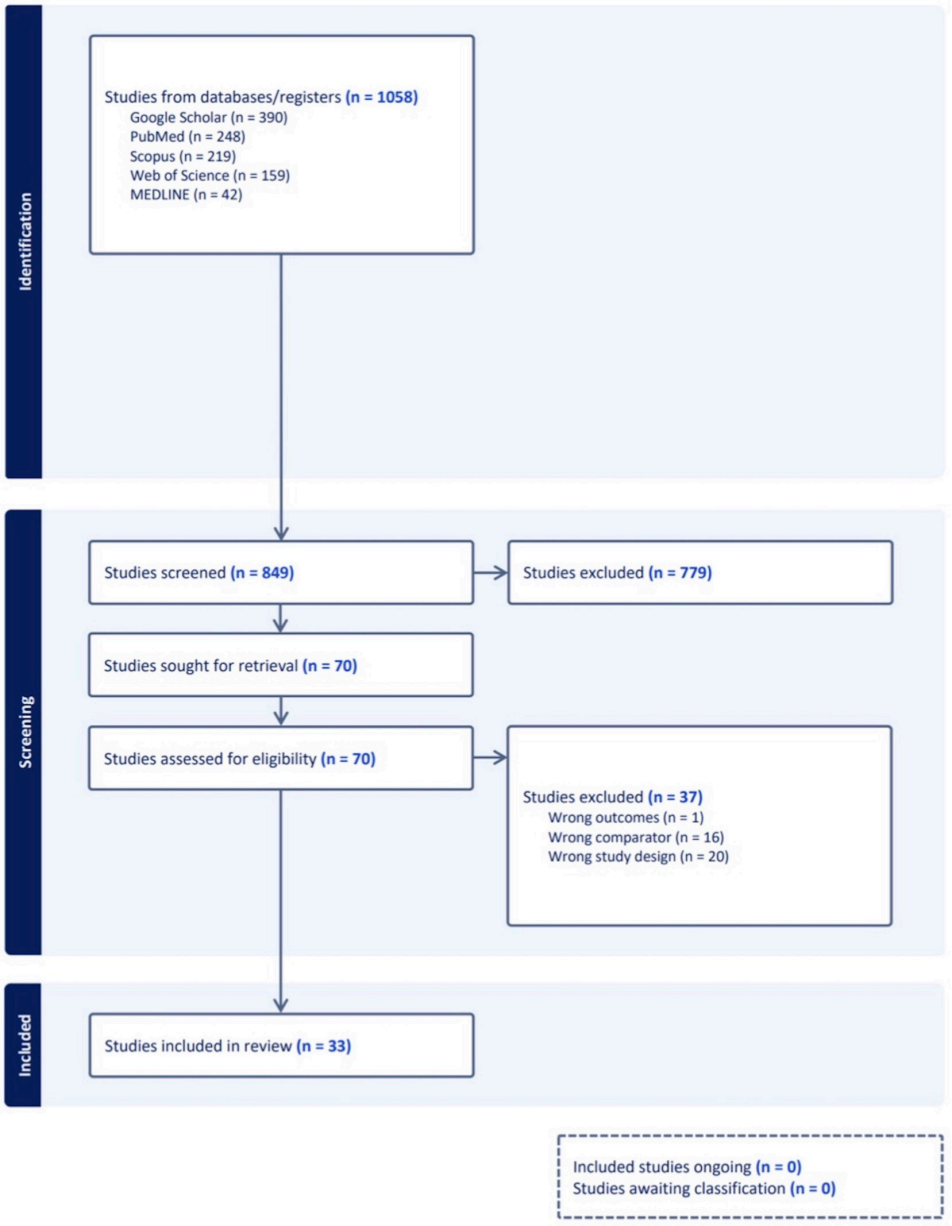
PRISMA flow chart showing inclusion and exclusion criteria. The diagram illustrates the process of study selection for this systematic review. Initially, 1058 studies were identified from various databases. In our post-screening and eligibility assessment, 779 studies were excluded for reasons such as wrong outcomes, wrong comparators, or inappropriate study design. Ultimately, 33 studies met inclusion criteria.

In this comprehensive review, 1,058 studies were screened, of which 33 studies from 16 countries met inclusion criteria (Fig 1). These studies were conducted between 1967 to 2020 and consisted of a diverse range of designs including 21 cross-sectional studies, nine case series, two cohort studies, and one case-control study (Table 1). All references included in this study represented a LOE III ‐ V. Study quality assessment from two reviewers yielded a high mean JBI score of 92.06% with a Cohen kappa score of 0.94, indicating high interrater reliability.

**Table 1:** Comprehensive overview of research studies.

Reference	LOE	Study Type	Patients w/ EEM	Organ^a^	Syndromic Data	Key Focus	Main Conclusion
Alexander 2020 [6]	IV	Cross sectional study	428	C	Yes	Cardiac abnormalities associated with microtia	Patients with microtia are at significant risk for cardiac abnormalities.
Aramaki 2006 [7]	IV	Case series	17	B	Yes	CHARGE Syndromes	Genomic testing for CHD7 provides health anticipatory guidance for patients with CHARGE syndrome.
Barbon 2022 [8]	IV	Cross sectional study	150	R	Yes	Renal abnormalities associated with isolated periauricular vestiges	Renal ultrasound screening is recommended for patients with isolated ear findings.
Barisic 2014 [9]	IV	Cross sectional study	259	B	Yes	Oculo-auriculo-vertebral spectrum	Early screening is warranted in patients OAV spectrum.
Beleza-Meireles 2015 [10]	IV	Cross sectional study	47	B	Yes	Oculo-auriculo-vertebral spectrum	Mutations of 22q11 is one of several possible causes of OAV spectrum.
Bellini 2001 [11]	IV	Case series	6	R	Yes	Ear and renal abnormalities in BOR Syndrome	BOR syndrome should be suspected in all cases of isolated urological anomalies.
Biard 2021 [12]	IV	Case series	10	C	Yes	CHARGE Syndromes	Fetuses with congenital heart defects should be carefully screened for CHARGE syndrome.
Cao 2021 [13]	IV	Cross sectional study	895	C	No	Cardiac abnormalities associated with microtia	Patients with microtia have a higher risk of congenital heart defects.
Davenport 1986 [14]	IV	Case series	14	B	Yes	CHARGE Syndromes	Severity of CHARGE syndrome features are highly variable with no single feature necessary for diagnosis.
Deshpande 2006 [15]	IV	Case control study	91	R	No	Renal abnormalities associated with external ear abnormalities	Renal imaging is not warranted in infants with minor external ear anomalies unless accompanied by other systemic malformations.
Digilio 2008 [16]	IV	Cross sectional study	87	B	Yes	Congenital heart disease and oculo-auriculo-vertebral spectrum	Congenital heart defects are significantly associated with OAV spectrum.
Firat 2008 [17]	IV	Cross sectional study	36	R	No	Renal abnormities associated with preauricular tags and pits	Renal imaging is not warranted in infants with isolated pre-auricular tags or pits.
Guo 2021 [18]	IV	Cross sectional study	804	C	No	Cardiac abnormalities associated with microtia	Patients with isolated microtia have a higher risk of congenital heart defects.
Kini 2020 [19]	IV	Cohort study	98	R	Yes	Renal abnormalities associated with microtia	Children with microtia are at a significant risk of structural renal abnormalities.
Koenig 2018 [20]	III	Cohort study	80	R	Yes	Renal abnormalities associated with microtia	Renal imagining is warranted in syndromic and non-syndromic patients with microtia.
Kohelet 2000 [21]	IV	Cross sectional study	70	R	No	Urinary tract abnormalities associated with Isolated preauricular tags	A significant prevalence of urinary tract abnormalities is detected in infants with preauricular tags.
Kugelman 2002 [22]	IV	Cross sectional study	92	R	No	Renal abnormalities associated with preauricular tags	Renal imaging is not warranted in infants with isolated pre-auricular tags or pits.
Llano-Rivas 1999 [23]	IV	Cross sectional study	145	R	No	Abnormalities associated with microtia	Early screening is warranted in patients with microtia.
Luquetti 2013 [24]	IV	Cross sectional study	818	B	No	Abnormal associations with microtia	Facial anomaly frequency increased with the severity of the microtia, while other anomalies are constant across all types of microtia.
Marfatia 2016 [25]	IV	Cross sectional study	30	B	Yes	Abnormalities associated with Anotia and Microtia	Congenital external malformations may be associated with spectrum of other anomalies.
Mishra 2003 [26]	IV	Cross sectional study	34	R	No	Renal abnormalities associated with preauricular tags	Patients with preauricular tags are at significant risk for cardiac abnormalities.
Morrison 1992 [27]	IV	Cross sectional study	25	C	Yes	Oculo-auriculo-vertebral spectrum	Care should be taken when electing to perform non-emergent surgery on OAV patients due to anesthesia risks.
Paul 2021 [28]	IV	Cross sectional study	694	B	No	Abnormalities associated with microtia	Comprehensive screening must be performed after a microtia diagnosis.
Ramprasad 2020 [29]	IV	Cross sectional study	98	B	No	Abnormal associations with microtia	Renal anomalies and cardiac defects are associated in patients with isolated microtia.
Rapin 1976 [30]	IV	Case series	16	B	No	Abnormal associations with ear malformation	Children with malformed ears may present with visceral anomalies.
Rashad 2007 [31]	IV	Case series	50	R	No	Renal abnormalities associated auricular malformations	Ear malformations are associated with an increased frequency of structural renal anomalies.
Salameh 2016 [32]	IV	Case series	64	R	No	Renal abnormalities associated with microtia	Pre- and post-natal screening for renal anomalies show the same level of detection.
Schrander-Stumpel 2005 [33]	IV	Cross Sectional Study	20	B	Yes	Kabuki Syndrome	Cardiac and renal abnormalities are found to be common in Kabuki syndrome.
Stoll 2016 [34]	IV	Cross sectional study	146	B	No	Abnormalities associated with anotia and microtia	High rates of anomalies are seen in individuals with microtia or anotia emphasizing the need for screening.
Strömland 2007 [35]	IV	Cross sectional study	18	C	Yes	Oculo-auriculo-vertebral spectrum	Study further elucidates systemic malformations seen in OAV spectrum.
Wang 2001 [36]	IV	Case series	42	R	Yes	Renal abnormalities associated with ear malformations	Ear malformations are associated with an increased frequency of renal anomalies.
Zhang 2018 [37]	IV	Case series	672	B	No	Abnormal associations with microtia	Congenital malformations are associated in a Chinese clinical population of patients with microtia.
Zim 2017 [38]	IV	Cross sectional study	514	R	No	Abnormal associations with microtia	Patients with isolated microtia have no increased prevalence of renal anomalies.

Comprehensive overview of selected 33 studies, including details such as the study reference, the level of evidence (LOE), study type, the number of patients involved with ear and external malformations (EEM), which associated organ anomaly was identified, along with the respective focus and key findings.

^a^ “R” denotes Renal, “C” denotes Cardiac, “B” denotes Both Renal and Cardiac

**Fig 2:**
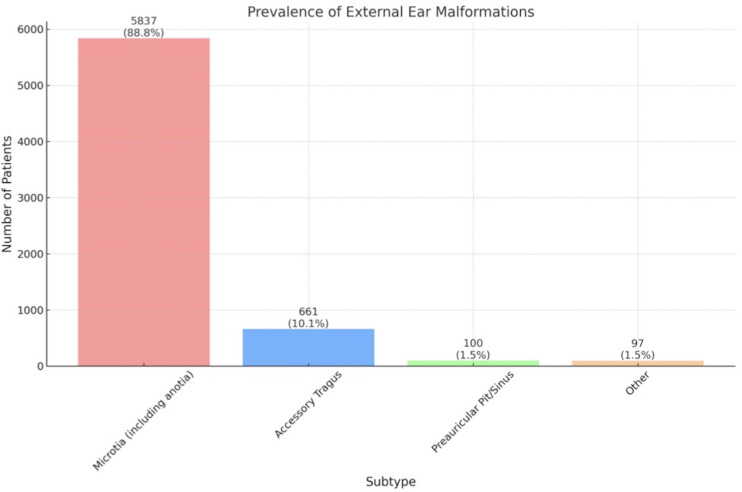
Prevalence diagram of EEM subtypes in this patient cohort. Microtia, including anotia, accounted for 88.8% of patients. Accessory tragus accounted for 10.1% of patients, while preauricular pit/sinus and other unspecified malformations accounted for 1.5% and 1.4% of patients, respectively. Note, a subset of patients presented with more than one malformation.

Of the 20,032 patients included, 6,570 EEM cases were identified. Among this cohort, the sex distribution was as follows: 3,558 individuals identified as male, 1,996 as female, and 1,017 patients were unspecified. Only six studies reported patient age (weighted mean age = 11.75; SD = 2.29). Ethnic demographics were detailed in two studies, revealing a predominance of Hispanic (71.97%) and White (16.30%) patients. A total of 5,837 patients were identified with microtia, which includes cases of anotia. Additionally, 661 patients presented with accessory tragus, 100 patients had preauricular pit/sinus, and 97 patients exhibited other forms of EEM (Fig 2). Several patients exhibited more than one type of EEM, indicating a prevalence of multiple ear malformations in some individuals. Sixteen of the 33 studies identified 718 patients with syndromic associations, a breakdown of EEM subtypes can be found in S1 Fig.

### Cardiac

Subset analysis of 20 studies that identified cardiac abnormalities was performed. Prior to pooling analysis, this subset of 5,243 patients with EEM contained 643 patients (12.26%) with various cardiac conditions. Of these patients, 464 individuals were diagnosed with unilateral or bilateral microtia, twelve were noted to have anotia, six patients had preauricular tags or pits, while the specific type of ear malformation in other cardiac presentations remained unspecified.

**Fig 3:**
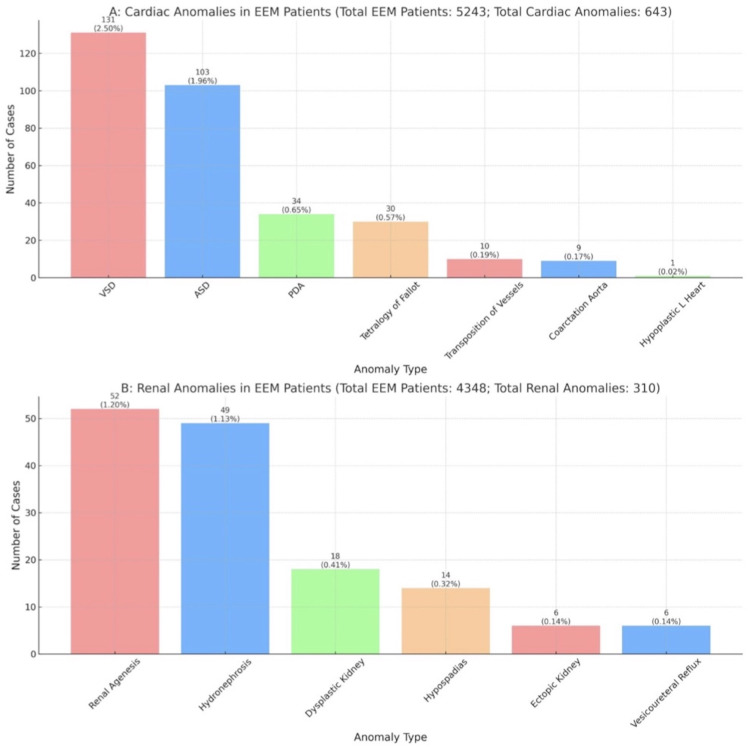
Cardiac and renal anomalies in EEM patients. Raw prevalence of cardiac and renal anomalies in patients with External Ear Malformations (EEM) as reported across 20 studies for cardiac anomalies (A) and 26 for renal anomalies (B).

Specific cardiac pathologies were identified in 498 (9.40%) cases of patients with EEM, of which the observed rates for these conditions were as follows: Ventricular Septal Defect (VSD) at 2.50%, Atrial Septal Defect (ASD) at 1.96%, Patent Ductus Arteriosus (PDA) at 0.65%, Tetralogy of Fallot at 0.57%, Transposition of Vessels at 0.19%, Coarctation of the Aorta at 0.17%, Hypoplastic Left Heart Syndrome at 0.02%, and other cardiac abnormalities at 3.34% (Fig 3A and S2 Table).

**Fig 4:**
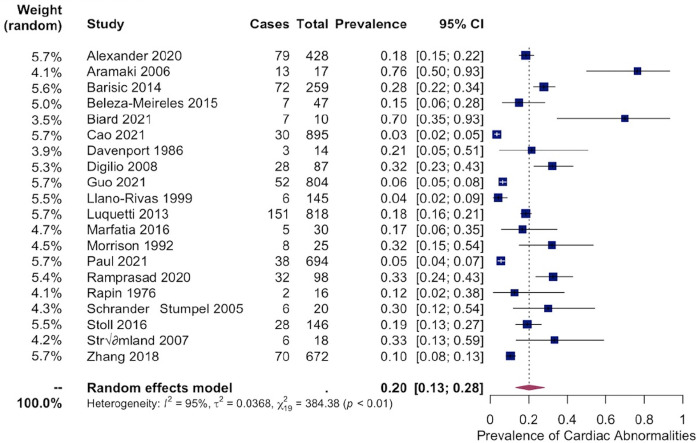
Pooled prevalence of cardiac abnormalities. Forest plot showing pooled prevalence of cardiac abnormalities among EEM patients from 20 studies. An overall pooled prevalence was 20% [95% CI:13–28%] was observed.

**Fig 5:**
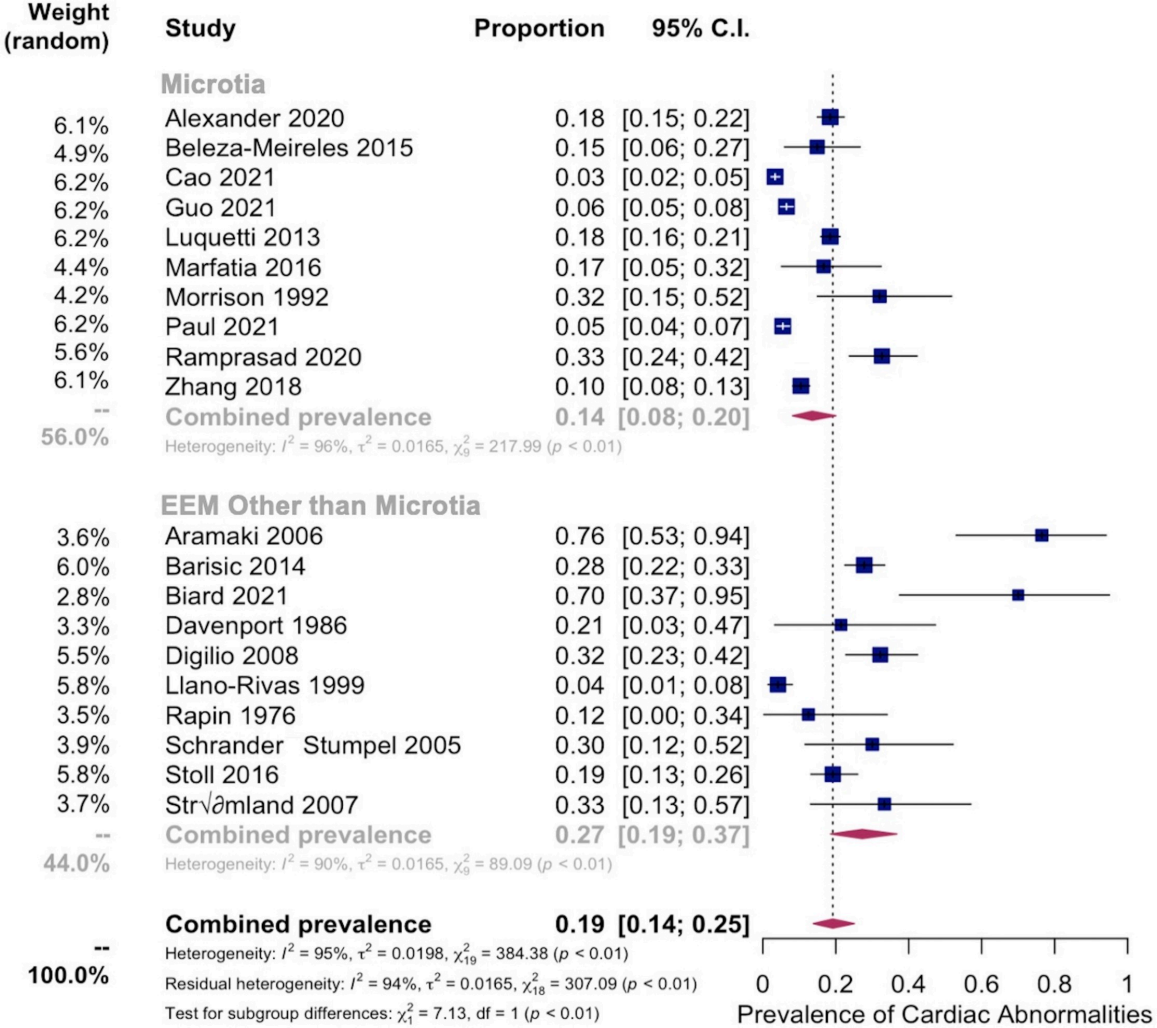
Cardiac subgroup prevalence analysis: Microtia vs Other EEM. Subgroup Analysis showing Pooled Prevalence of Cardiac Abnormalities in Microtia vs Other EEM. (Top) Pooled prevalence of cardiac abnormalities in microtia from 10 studies. (Bottom) Pooled prevalence of cardiac abnormalities in other EEM from 10 studies.

Meta-analyses were then performed on these 20 studies investigating the pooled prevalence of cardiac abnormalities in EEM patients. The overall pooled prevalence was 20% [95% CI:13–28%] (Fig 4). A sub-analysis comparing microtia to other forms of EEMs showed a pooled prevalence of 14% [95% CI: 8–20%] in patients with microtia and 27% [95% CI: 19–37%] in patients with other types of EEMs. Heterogeneity in all models was high (I2 = 95% for both studies) (Fig 5). Moderator testing considering factors such as year of study and sample size did not yield significant results. Similarly, moderator testing of the presence of syndrome data was insignificant. Supplementary conservative meta-analyses, excluding outlier studies that significantly affected the overall summary proportion, were also conducted (S2 Fig).

### Renal

A subset analysis of 26 studies with identified renal abnormalities was performed. Prior to pooling analysis, this subset included 4348 patients, of which 301 patients (7.13%) were observed to have various renal conditions. Among these patients, 147 cases involved unilateral or bilateral microtia, 4 patients had documented anotia, 20 cases had an accessory tragus, six cases included patients with pre-auricular pits, while the remaining patients with renal abnormalities had unspecified EEM.

Specific renal pathologies were identified in 248 (5.70%) cases of patients with EEM, of which the observed rates for these conditions were as follows: Renal Agenesis at 1.20%, Hydronephrosis at 1.13%, Multicystic Dysplastic kidneys at 0.41%, Hypospadias at 0.37%, Ectopic kidney at 0.14%, Vesicoureteral Reflux at 0.14%, and other classified cardiac abnormalities at 2.37% (Fig 3B and S2 Table).

**Fig 6:**
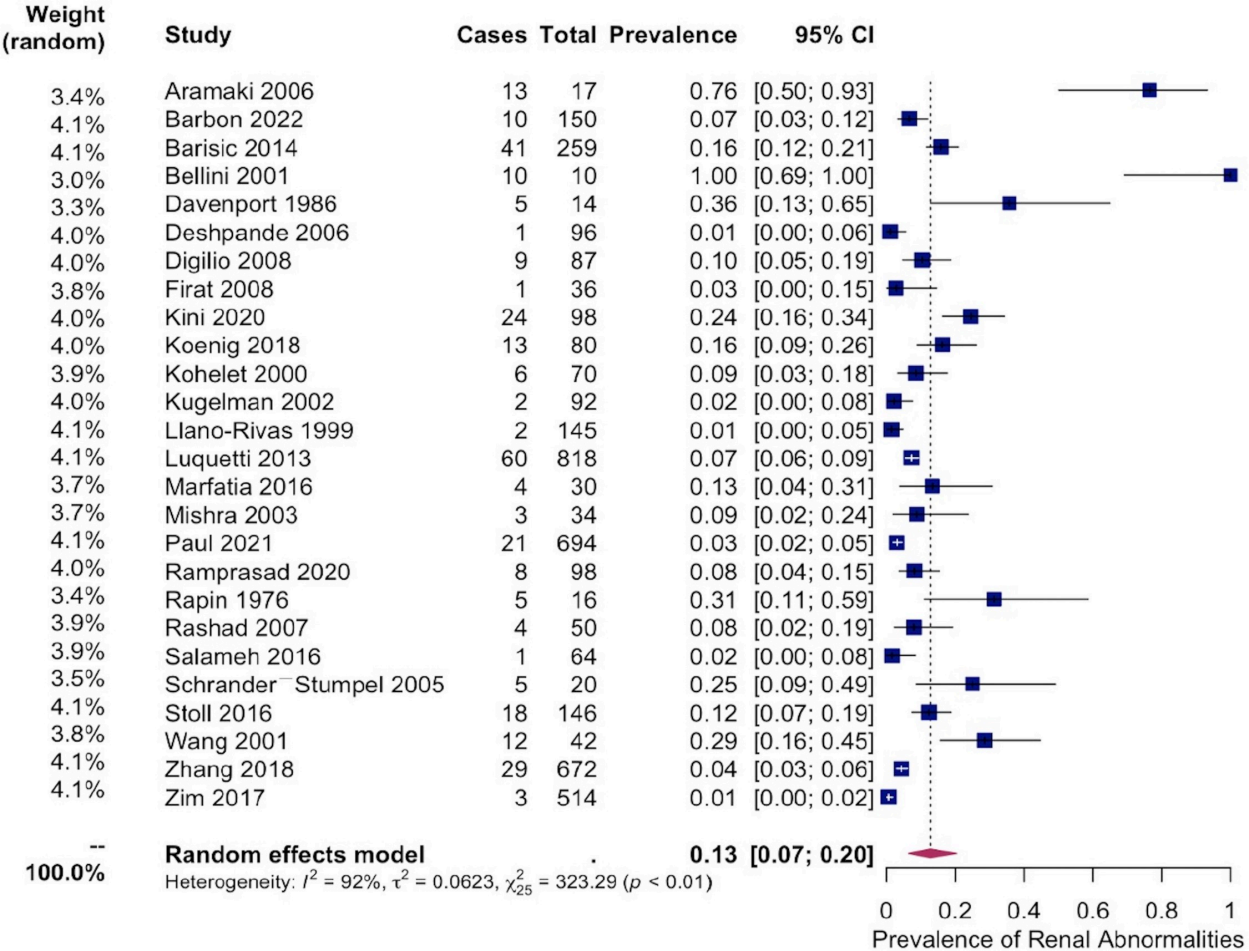
Pooled prevalence of renal abnormalities. Forest plot showing pooled prevalence of renal abnormalities among EEM patients from 26 studies. An overall prevalence of 13% [95% CI: 7–20%] between EEM and renal abnormalities was observed.

**Fig 7:**
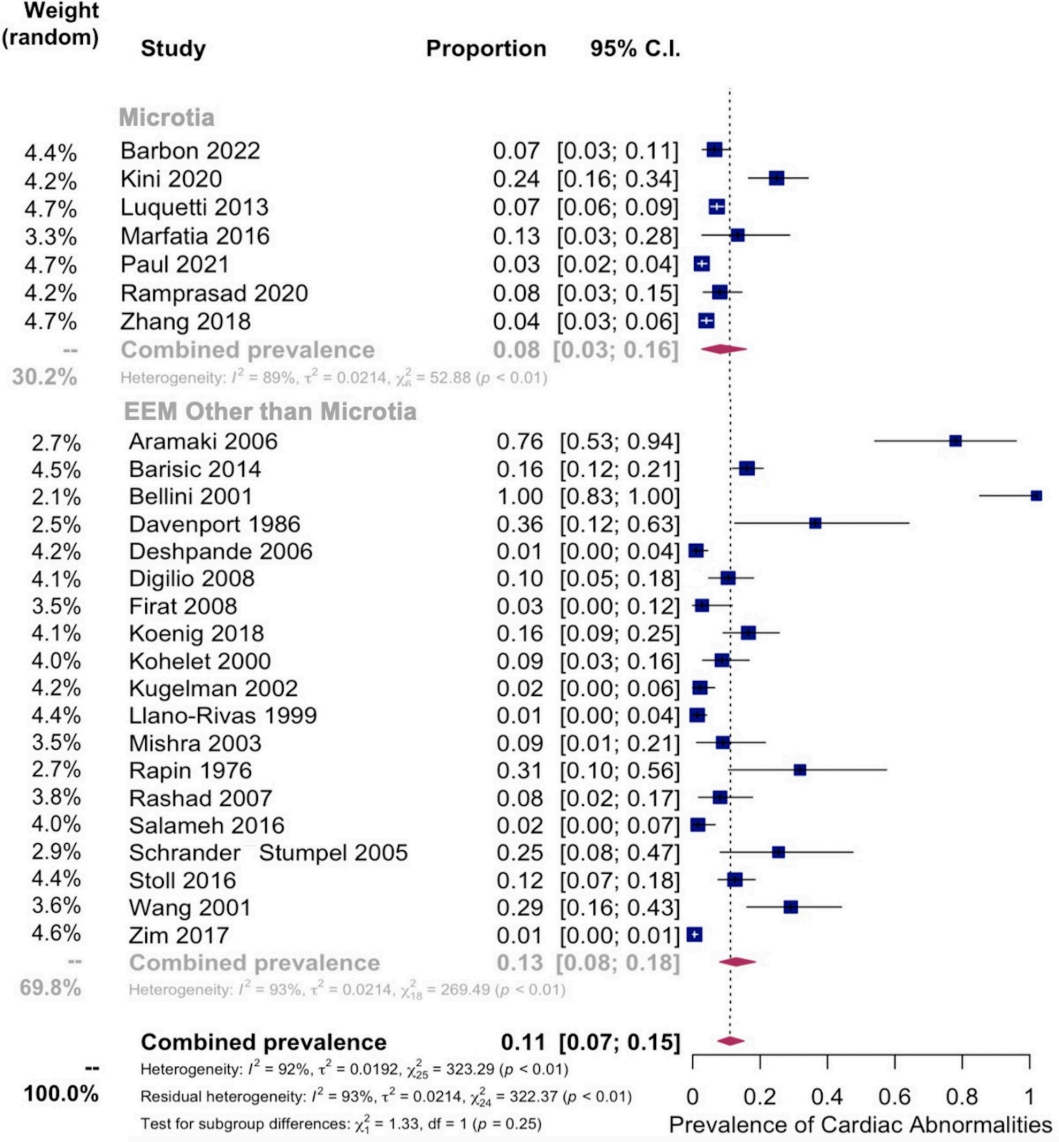
Renal subgroup prevalence analysis: Microtia vs Other EEM. Subgroup Analysis showing Pooled Prevalence of Renal Abnormalities in Microtia vs Other EEM. (Top) Pooled prevalence of renal abnormalities in microtia from 7 studies. (Bottom) Pooled prevalence of renal abnormalities in other EEM from 19 studies.

Meta-analysis was then performed on 26 studies investigating the pooled prevalence of renal abnormalities in EEM patients, which demonstrated an overall prevalence of 13% [95% CI: 7–20%] between EEM and renal abnormalities (Fig 6). A sub-analysis was performed comparing microtia to all other EEM’s, which demonstrated a pooled prevalence of renal abnormalities of 8% [95% CI: 3–16%] in patients with microtia and 13% [95% CI: 8–18%] in patients with all other EEM’s (Fig 7). Heterogeneity in all models was high (I^2^ = 92% percent in both models). Moderator testing again did not yield any significant results, and supplementary conservative meta-analyses were conducted excluding outlying studies with significant effects on the overall summary proportion (S2 Fig).

Given the higher incidence of cardiac over renal anomalies in patients with EEM, an additional sub-analysis of the thirteen studies that reported on both cardiac and renal outcomes was performed. This analysis demonstrated a pooled prevalence of cardiac and renal abnormalities in 21% and 14% of cases [95% CI:12–30%], respectively (S3 Fig).

## Discussion

External ear malformations are encountered in approximately 1 in 3800 live births [1]. Their association with cardiac and renal anomalies has been recognized primarily in the context of syndromic presentations, such as CHARGE syndrome, Townes-Brocks syndrome, branchio-oto-renal syndrome, Nager syndrome, Miller syndrome, and diabetic embryopathy [36]. This systematic review consolidated knowledge on the broader associations between EEM and cardiac/renal abnormalities, thereby augmenting the clinical understanding necessary for managing EEM sequelae. Although previous literature has described syndrome-specific EEM associations [7, 9–12, 16], discussions on the link between EEM and cardiac or renal abnormalities in a non-syndromic context are scant. Our review fills this gap by demonstrating a notable association between EEM and such malformations, regardless of syndromic conditions, which has direct implications for patient care and screening protocols. Importantly, our results found a higher rate of cardiac anomalies than renal anomalies in those with external ear malformations, highlighting the need to emphasize comprehensive screening in this unique patient population.

In this review, the pooled prevalence of cardiac versus renal abnormalities in EEM patients was 20% and 13%, respectively. This observation lies in contrast to the more common emphasis placed on renal anomalies in patients with EEM, which has led to widespread advocacy for renal ultrasound as a routine screening measure in these patients [19–21, 25, 26, 30, 35]. Conversely, the discourse on cardiac screening remains disproportionally limited, typically highlighted only within the context of specific syndromic conditions [9]. Furthermore, this study’s observation that cardiac anomalies appeared to be more common than renal anomalies was consistent when exploring studies that identified both cardiac and renal anomalies.

Our review confirms a pooled prevalence of cardiac anomalies in patients with EEM, with both ventricular and atrial septal defects being the most reported anomalies. This association was not confined to syndromic cases, such as CHARGE and branchio-oto-renal syndrome [9, 14, 24, 27], challenging the clinical perception that primarily associates these abnormalities with specific syndromes [6, 18, 23, 33, 34, 37]. The lack of data granularity across included studies did not support the ability to perform a sub-meta-analysis to determine if patients with a syndrome diagnosis had similar rates to patients without syndromes. However, our findings revealed that the moderator effect of recorded syndrome data was not statistically significant, indicating that the variability in prevalence rates of cardiac and renal anomalies cannot be explained by whether associated syndrome data were recorded or not. This implies that the presence of syndromes may not be a significant modifier of the relationship between EEM and cardiac or renal anomalies. In other words, the results imply a consistent association between EEM and cardiac and renal anomalies that is not significantly modulated by the presence of syndromic conditions across the studies. Thus, while syndromic conditions are clinically relevant markers to prompt further screening of patients, cardiac and renal abnormalities can persist outside of these syndromes and reinforce the need for vigilant screening in all patients with EEM.

This review indicates that the most common EEM with a prevalent cardiac abnormality was microtia. In this population, VSD and ASD were again the most common cardiac abnormalities noted, followed by PDA, Tetralogy of Fallot, and vessel transposition, which underscores the need for vigilant cardiac screening in EEM patients. Similarly, a pooled prevalence of renal abnormalities in EEM patients was observed, with renal agenesis and hydronephrosis reported most frequently. Twenty-six of the 33 studies included in this review reported renal abnormalities in patients with EEM, both in syndromic and non-syndromic patients. As with cardiac abnormalities, the most common EEM with a renal anomaly was microtia. As previously discussed, the literature frequently emphasizes renal ultrasound in EEM patients, reflecting the established discourse on the EEM-renal connection. This review corroborates these findings and reinforces the clinical imperative for renal screening, especially in cases with microtia, which bear a significant risk for such anomalies.

This study is not without limitations. This study is constrained by heterogeneity in study methodologies and reporting standards, which complicates the synthesis of data and interpretation of specific syndromic versus non-syndromic associations. Such disparities in reporting methods preclude accurate sub-analysis of specific syndromes. The variability in severity reporting further obscures the clinical relevance of the EEM-associated abnormalities. For example, treatment for VSD varies widely and can range from simple monitoring to surgical repair [39]. Without data on the severity of the associated abnormalities, it is difficult to correlate the clinical significance and identify the true needs for revised screening protocols.

This study is also at risk for inclusion bias due to the calculations of pooled prevalence confined only to those included studies. Lastly, the inconsistent inclusion of demographic data restricts the contextual understanding of EEM prevalence across different populations. Notwithstanding these limitations, our review demonstrates more insight into the prevalence between EEM and cardiac and renal anomalies, advocating for more granular research through large cohort studies employing standardized reporting criteria for abnormalities, their severity, and patient demographics.

## Conclusion

This systematic review and meta-analysis rigorously examined the existing literature to delineate the incidence of renal and cardiac anomalies associated with external ear malformations. Findings confirmed a significant prevalence between EEM and congenital cardiac and renal anomalies in syndromic and non-syndromic pediatric patients. The prevalence of cardiac anomalies exceeded that of renal anomalies, a finding that not only compels a reevaluation of current screening practices but also emphasizes the need for a more inclusive and balanced screening approach. This review provides a comprehensive synthesis of the associations between EEM and cardiac and renal anomalies, reinforcing the critical need for heightened awareness and targeted screening protocols to optimize patient outcomes in this patient population.

## Supporting information

S1 Fig Prevalence of external ear malformations in syndromic patients. This bar chart illustrates the prevalence of different subtypes of External Ear Malformations (EEM) in patients identified with syndromic conditions. The majority of patients are affected by microtia, including anotia, which constitutes 77.2% of cases. Accessory tragus malformations are present in 5.0% of patients, preauricular pit/sinus in 1.4%, and other EEM subtypes constitute 5.3% of the cases. Of note, a subset of patients presented with more than one malformation.
(DOCX)

S2 Fig Pooled prevalence of cardiac and renal abnormalities without outliers. Outliers that had significant effects on the overall summary proportion were excluded during supplemental conservative meta-analyses. A) Forest plot showing the prevalence of cardiac abnormalities in patients with EEM from 19 studies. An overall pooled prevalence was 18% [95% CI:12–24%] was observed. B) (Top) Pooled prevalence of cardiac abnormalities in microtia from ten studies. (Bottom) Pooled prevalence of cardiac abnormalities in EEM other than Microtia from 9 studies. A pooled prevalence of 14% [95% CI: 8–20%] in microtia patients and 23% [95% CI: 15–33%] in patients with other types of EEMs was observed. C) Forest plot showing pooled prevalence of renal abnormalities among EEM patients from 24 studies. An overall prevalence of 8% [95% CI: 5–12%] between EEM and renal abnormalities was observed. D) (Top) Pooled prevalence of renal abnormalities in microtia from 7 studies. (Bottom) Pooled prevalence of renal abnormalities in EEM other than Microtia from 17 studies. A pooled prevalence of 8% [95% CI: 4–14%] in microtia patients and 8% [95% CI: 5–13%] in patients with other types of EEMs.
(DOCX)

S3 Fig Prevalence of cardiac or renal abnormalities with EEM. Forest plot showing pooled prevalence of cardiac A) and renal B) abnormalities among EEM patients from 11 studies that reported both cardiac and renal anomalies. An overall pooled prevalence of cardiac anomalies was seen in 21% of cases [95% CI:12–30%], while an overall pooled prevalence of renal anomalies was seen in 14% of cases [95% CI:6–24%].
(DOCX)

S1 Table Search string. Comprehensive List of Databases Consulted, Corresponding Search Queries, and the Resulting Number of Studies Retrieved.
(DOCX)

S2 Table EEM and associated anomalies. External Ear Malformations (EEM) and associated anomalies in patients from 20 studies focused on cardiac irregularities and 26 studies on renal anomalies. A total of 5,243 patients with EEM were reviewed for cardiac anomalies, of which 643 patients (12.26%) were identified, while a total of 4348 patients with EEM were reviewed for renal anomalies, of which 310 patients (7.13%) were identified.
(DOCX)

S1 Data Included and excluded papers. (CSV)

S2 Data Quality assessment. (XLSX)

S3 Data Raw data. (CSV)

S1 Checklist PRISMA 2020 checklist. (DOCX)
