# Supplementary figures and images for: External ear malformations and cardiac and renal anomalies: A systematic review and meta-analysis
Source: PLoS One. 2024 Sep 19;19(9):e0309692. doi: 10.1371/journal.pone.0309692 (PMC11412664; doi:10.1371/journal.pone.0309692)

**S1 Fig: Prevalence of External Ear Malformations in Syndromic Patients.**
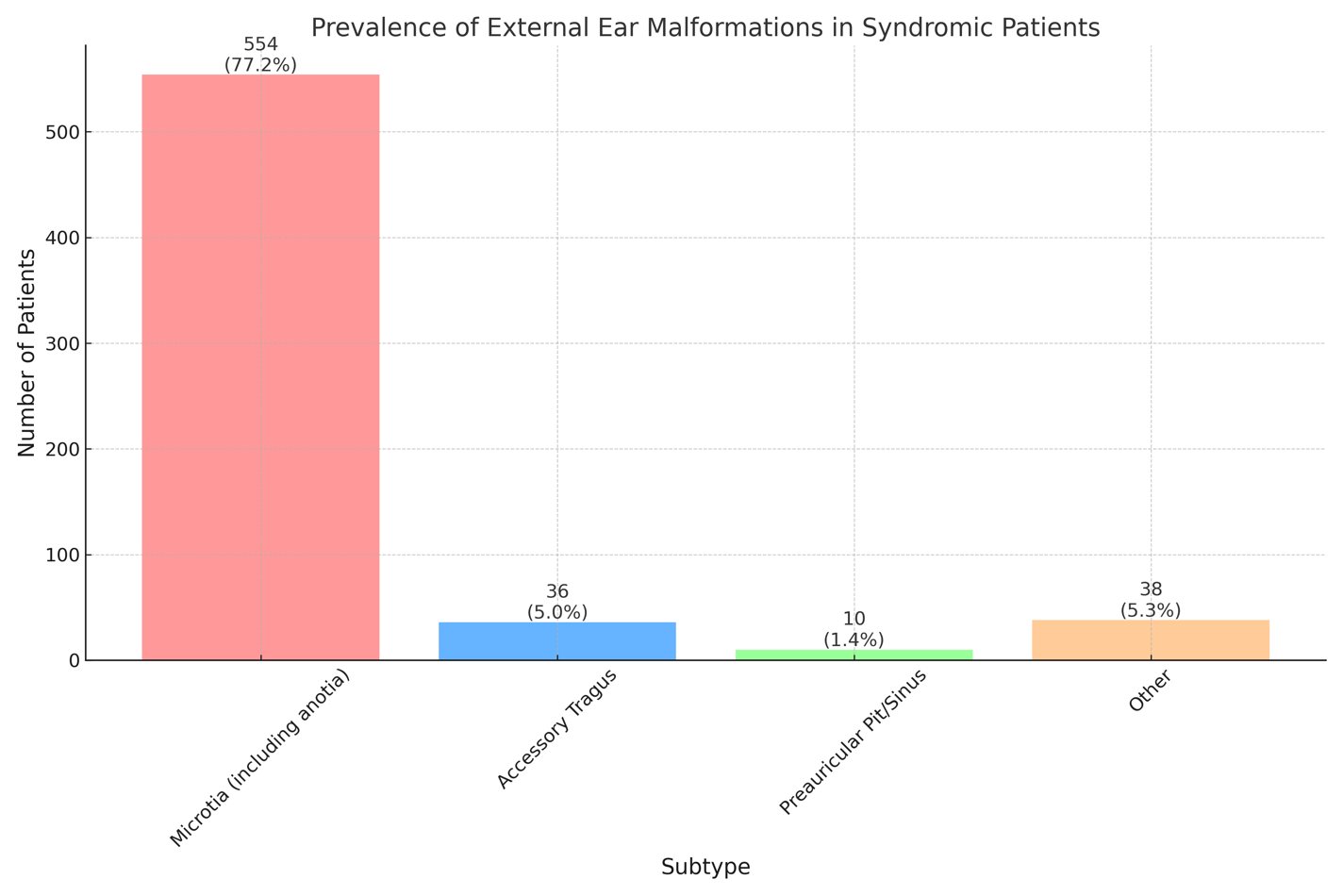

Supplement: S1 Fig — This bar chart illustrates the prevalence of different subtypes of External Ear Malformations (EEM) in patients identified with syndromic conditions. The majority of patients are affected by microtia, including anotia, which constitutes 77.2% of cases. Accessory tragus malformations are present in 5.0% of patients, preauricular pit/sinus in 1.4%, and other EEM subtypes constitute 5.3% of the cases. Of note, a subset of patients presented with more than one malformation. (DOCX) [file pone.0309692.s001.docx]

**S2 Fig. Pooled Prevalence of Cardiac and Renal Abnormalities without Outliers.**
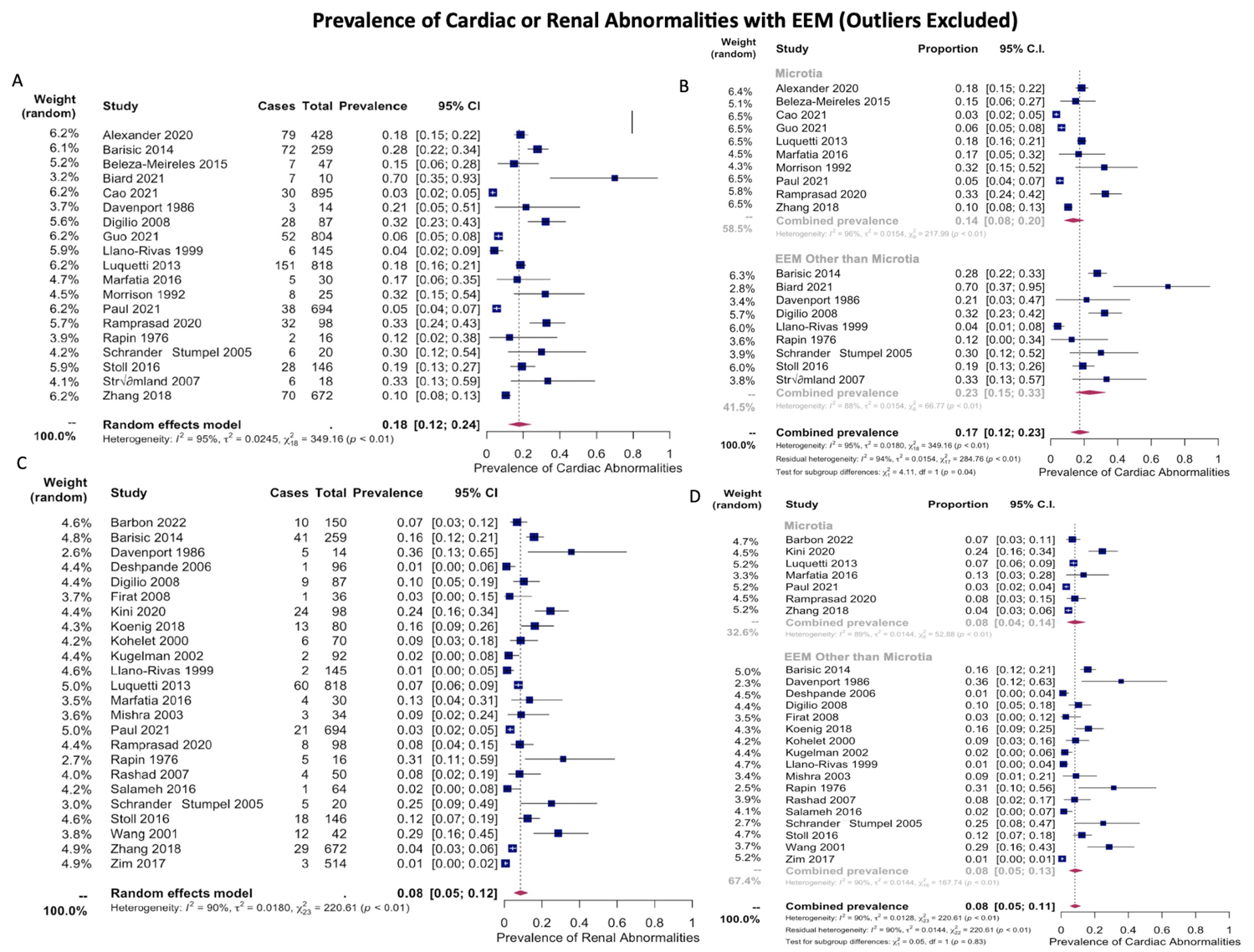

Supplement: S2 Fig — Outliers that had significant effects on the overall summary proportion were excluded during supplemental conservative meta-analyses. A) Forest plot showing the prevalence of cardiac abnormalities in patients with EEM from 19 studies. An overall pooled prevalence was 18% [95% CI:12–24%] was observed. B) (Top) Pooled prevalence of cardiac abnormalities in microtia from ten studies. (Bottom) Pooled prevalence of cardiac abnormalities in EEM other than Microtia from 9 studies. A pooled prevalence of 14% [95% CI: 8–20%] in microtia patients and 23% [95% CI: 15–33%] in patients with other types of EEMs was observed. C) Forest plot showing pooled prevalence of renal abnormalities among EEM patients from 24 studies. An overall prevalence of 8% [95% CI: 5–12%] between EEM and renal abnormalities was observed. D) (Top) Pooled prevalence of renal abnormalities in microtia from 7 studies. (Bottom) Pooled prevalence of renal abnormalities in EEM other than Microtia from 17 studies. A pooled prevalence of 8% [95% CI: 4–14%] in microtia patients and 8% [95% CI: 5–13%] in patients with other types of EEMs. (DOCX) [file pone.0309692.s002.docx]

**S3 Fig. Prevalence of Cardiac or Renal Abnormalities with EEM**
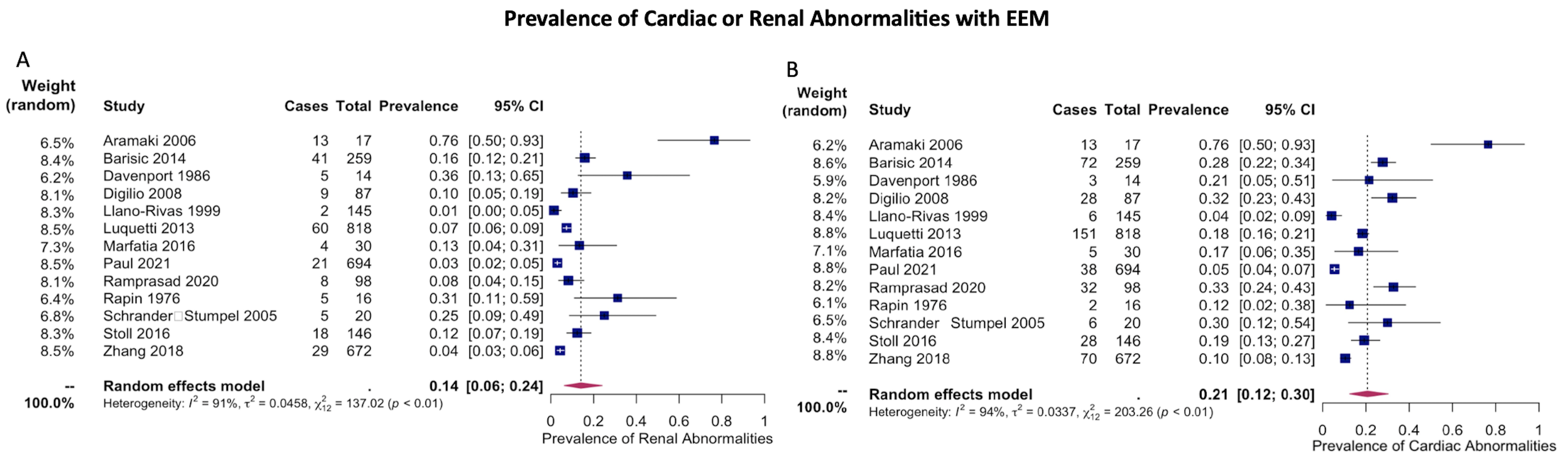

Supplement: S3 Fig — Forest plot showing pooled prevalence of cardiac A) and renal B) abnormalities among EEM patients from 11 studies that reported both cardiac and renal anomalies. An overall pooled prevalence of cardiac anomalies was seen in 21% of cases [95% CI:12–30%], while an overall pooled prevalence of renal anomalies was seen in 14% of cases [95% CI:6–24%]. (DOCX) [file pone.0309692.s003.docx]
