# Supplementary material for: External ear malformations and cardiac and renal anomalies: A systematic review and meta-analysis
Source: PLoS One. 2024 Sep 19;19(9):e0309692. doi: 10.1371/journal.pone.0309692 (PMC11412664; doi:10.1371/journal.pone.0309692)
Supplement: S1 Table — Comprehensive List of Databases Consulted, Corresponding Search Queries, and the Resulting Number of Studies Retrieved. (DOCX) [file pone.0309692.s004.docx]

**S1 Table. Search String.** Comprehensive List of Databases Consulted, Corresponding Search Queries, and the Resulting Number of Studies Retrieved

| Database | Search String |  |
| --- | --- | --- |
| PubMed | ("Congenital Microtia"[MeSH] OR "Ear/abnormalities"[MeSH] OR "Accessory Tragus"[tiab] OR Microtia*[tiab] OR Anotia*[tiab] OR "Preauricular Pits"[tiab] OR Cryptotia*[tiab] OR "Protruding Ears"[tiab] OR "Constricted Ears"[tiab] OR "Stahl’s Ear"[tiab] OR "Earlobe deformit*"[tiab] OR "Ear hemangiomas"[tiab] OR "Ear Lidding Deformity"[tiab] OR "Helical Rim Ear Deformity"[tiab] OR "Conchal Crus Ear Deformity"[tiab]) AND    (("Heart Defects, Congenital"[MeSH] OR "Tetralogy of Fallot"[tiab] OR "Pulmonary Stenosis"[tiab] OR "Overriding Aorta"[tiab] OR "Ventricular Hypertroph*"[tiab] OR Cardiomegal*[tiab] OR ASD[tiab] OR VSD[tiab] OR PFO[tiab] OR "Ebstein anomal*"[tiab] OR "Transposition of the great arter*"[tiab] OR "Coarctation of the aorta"[tiab]) OR    ("Congenital urologic anomal*"[tiab] OR "Renal abnormalit*"[tiab] OR "Bilateral renal agenesis"[tiab] OR "Ectopic kidney"[tiab] OR "Multicystic dysplastic kidney"[tiab] OR "Duplicated collecting system"[tiab] OR VUR[tiab] OR Hydronephros*[tiab] OR "Renal hypoplasia"[tiab] OR Ureterocele*[tiab] OR Megaureter*[tiab])) | 248 |
| MEDLINE | ("Congenital Microtia" OR "Ear/abnormalities" OR "Accessory Tragus" OR Microtia*.ti,ab OR Anotia*.ti,ab OR "Preauricular Pits".ti,ab OR Cryptotia*.ti,ab OR "Protruding Ears".ti,ab OR "Constricted Ears".ti,ab OR "Stahl’s Ear".ti,ab OR "Earlobe deformit*".ti,ab OR "Ear hemangiomas".ti,ab OR "Ear Lidding Deformity".ti,ab OR "Helical Rim Ear Deformity".ti,ab OR "Conchal Crus Ear Deformity".ti,ab) AND (("Heart Defects, Congenital" OR "Tetralogy of Fallot".ti,ab OR "Pulmonary Stenosis".ti,ab OR "Overriding Aorta".ti,ab OR "Ventricular Hypertroph*".ti,ab OR Cardiomegal*.ti,ab OR ASD.ti,ab OR VSD.ti,ab OR PFO.ti,ab OR "Ebstein anomal*".ti,ab OR "Transposition of the great arter*".ti,ab OR "Coarctation of the aorta".ti,ab) OR ("Congenital urologic anomal*".ti,ab OR "Renal abnormalit*".ti,ab OR "Bilateral renal agenesis".ti,ab OR "Ectopic kidney".ti,ab OR "Multicystic dysplastic kidney".ti,ab OR "Duplicated collecting system".ti,ab OR VUR.ti,ab OR Hydronephros*.ti,ab OR "Renal hypoplasia".ti,ab OR Ureterocele*.ti,ab OR Megaureter*.ti,ab)) | 42 |
| Web of Science | (TS=("Congenital Microtia" OR "Ear abnormalities" OR "Accessory Tragus" OR Microtia* OR Anotia* OR "Preauricular Pits" OR Cryptotia* OR "Protruding Ears" OR "Constricted Ears" OR "Stahl’s Ear" OR "Earlobe deformit*" OR "Ear hemangiomas" OR "Ear Lidding Deformity" OR "Helical Rim Ear Deformity" OR "Conchal Crus Ear Deformity") AND (TS=("Heart Defects, Congenital" OR "Tetralogy of Fallot" OR "Pulmonary Stenosis" OR "Overriding Aorta" OR "Ventricular Hypertroph*" OR Cardiomegal* OR ASD OR VSD OR PFO OR "Ebstein anomal*" OR "Transposition of the great arter*" OR "Coarctation of the aorta") OR TS=("Congenital urologic anomal*" OR "Renal abnormalit*" OR "Bilateral renal agenesis" OR "Ectopic kidney" OR "Multicystic dysplastic kidney" OR "Duplicated collecting system" OR VUR OR Hydronephros* OR "Renal hypoplasia" OR Ureterocele* OR Megaureter*))) | 159 |
| Scopus | (TITLE-ABS-KEY ("Congenital Microtia" OR "Ear abnormalities" OR "Accessory Tragus" OR Microtia* OR Anotia* OR "Preauricular Pits" OR Cryptotia* OR "Protruding Ears" OR "Constricted Ears" OR "Stahl’s Ear" OR "Earlobe deformit*" OR "Ear hemangiomas" OR "Ear Lidding Deformity" OR "Helical Rim Ear Deformity" OR "Conchal Crus Ear Deformity") AND    TITLE-ABS-KEY ("Heart Defects, Congenital" OR "Tetralogy of Fallot" OR "Pulmonary Stenosis" OR "Overriding Aorta" OR "Ventricular Hypertroph*" OR Cardiomegal* OR ASD OR VSD OR PFO OR "Ebstein anomal*" OR "Transposition of the great arter*" OR "Coarctation of the aorta") OR TITLE-ABS-KEY ("Congenital urologic anomal*" OR "Renal abnormalit*" OR "Bilateral renal agenesis" OR "Ectopic kidney" OR "Multicystic dysplastic kidney" OR "Duplicated collecting system" OR VUR OR Hydronephros* OR "Renal hypoplasia" OR Ureterocele* OR Megaureter*)) | 219 |
| GoogleScholar | ("Ear malformations") AND (("Heart Defects, Congenital" OR "Tetralogy of Fallot" OR "Pulmonary Stenosis" OR Cardiomegaly OR ASD OR VSD) AND    ("Congenital urologic anomalies" OR "Renal abnormalities" OR "Bilateral renal agenesis")) | 390 |
| Total articles screened: 1058 | | |
