# Supplementary material for: External ear malformations and cardiac and renal anomalies: A systematic review and meta-analysis
Source: PLoS One. 2024 Sep 19;19(9):e0309692. doi: 10.1371/journal.pone.0309692 (PMC11412664; doi:10.1371/journal.pone.0309692)
Supplement: S2 Table — External Ear Malformations (EEM) and associated anomalies in patients from 20 studies focused on cardiac irregularities and 26 studies on renal anomalies. A total of 5,243 patients with EEM were reviewed for cardiac anomalies, of which 643 patients (12.26%) were identified, while a total of 4348 patients with EEM were reviewed for renal anomalies, of which 310 patients (7.13%) were identified. (DOCX) [file pone.0309692.s005.docx]

**S2 Table.** **EEM and Associated Anomalies** External Ear Malformations (EEM) and associated anomalies in patients from 20 studies focused on cardiac irregularities and 26 studies on renal anomalies.

| **EEM Cases in 20 Studies Reporting Cardiac Anomalies** | 5243 patients |
| --- | --- |
| **Cardiac Abnormality Patients** | **643 (12.26%)** |
| **Specified Cardiac Anomalies Cases** | **493 (9.40%)** |
| VSD | 131 (2.50%) |
| ASD | 103 (1.96%) |
| PDA | 34 (0.65%) |
| Tetralogy of Fallot | 30 (0.57%) |
| Transposition of Vessels | 10 (0.19%) |
| Coarctation Aorta | 9 (0.17%) |
| Hypoplastic L Heart | 1 (0.02%) |
| Other Cardiac | 175 (3.34%) |
| **EEM Cases in 26 Studies Reporting Renal Anomalies** | 4348 patients |
| **Kidney/urogenital Abnormality Patients** | **310 (7.13%)** |
| **Specified Renal Anomalies Cases** | **248 (5.70%)** |
| Renal Agenesis | 52 (1.20%) |
| Hydronephrosis | 49 (1.13%) |
| Dysplastic Kidney | 18 (0.41%) |
| Hypospadias | 14 (0.37%) |
| Ectopic Kidney | 6 (0.14%) |
| Vesicoureteral Reflux | 6 (0.14%) |
| Other Renal/Urogenital | 103 (2.37%) |
